# Fish Collagen: Extraction, Characterization, and Applications for Biomaterials Engineering

**DOI:** 10.3390/polym12102230

**Published:** 2020-09-28

**Authors:** Hafez Jafari, Alberto Lista, Manuela Mafosso Siekapen, Pejman Ghaffari-Bohlouli, Lei Nie, Houman Alimoradi, Amin Shavandi

**Affiliations:** 1 BioMatter Unit—BTL, École Polytechnique de Bruxelles, Université Libre de Bruxelles, Avenue F.D. Roosevelt, 50-CP 165/61, 1050 Brussels, Belgium; 2 Department of Chemistry, Materials and Chemical Engineering “Giulio Natta”, Politecnico di Milano, Piazza Leonardo da Vinci, 32, 20133 Milan, Italy; Alberto.Lista@vub.be; 3 Department of Chemical Engineering and Industrial Chemistry, Vrije Universiteit Brussel, Boulevard de la Plaine 2, 1050 Brussels, Belgium; Manuela.Mafosso.Siekapen@vub.be; 4 Nano-Biopolymers Research Laboratory, School of Chemical Engineering, College of Engineering, University of Tehran, Tehran 11155-4563, Iran; pejman.ghaffari@ut.ac.ir; 5 College of Life Sciences, Xinyang Normal University, Xinyang 464000, China; 6 School of Biomedical Sciences, University of Otago, Dunedin 9016, New Zealand; houman.alimoradi@otago.ac.nz

**Keywords:** fish collagen, deep eutectic solvent, medical applications, collagen extraction

## Abstract

The utilization of marine-based collagen is growing fast due to its unique properties in comparison with mammalian-based collagen such as no risk of transmitting diseases, a lack of religious constraints, a cost-effective process, low molecular weight, biocompatibility, and its easy absorption by the human body. This article presents an overview of the recent studies from 2014 to 2020 conducted on collagen extraction from marine-based materials, in particular fish by-products. The fish collagen structure, extraction methods, characterization, and biomedical applications are presented. More specifically, acetic acid and deep eutectic solvent (DES) extraction methods for marine collagen isolation are described and compared. In addition, the effect of the extraction parameters (temperature, acid concentration, extraction time, solid-to-liquid ratio) on the yield of collagen is investigated. Moreover, biomaterials engineering and therapeutic applications of marine collagen have been summarized.

## 1. Introduction

**Figure 1:**
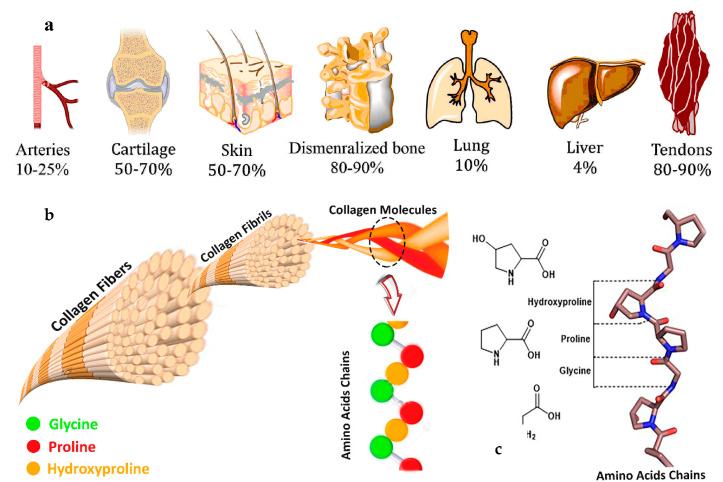
(**a**) Approximate content of collagen in different tissues; values are from https://www.elsevier.com/es-es/connect/medicina/colagenos-tipos-composicion-distribucion-tejidos. (**b**) Structure of collagen fibers, fibrils, triple helices of alpha chains and amino acid residues, 4-hydroxyproline (Hyp), glycine (Gly), and proline. (**c**) Amino acids chains structure of collagen.

Collagen is the most abundant structural protein in the extracellular matrix of the various connective tissues in the body (i.e., skin, bones, ligaments, tendons, and cartilage) (Figure 1a) [1,2]. The primary biomedical applications of collagen were in biomaterials, especially as drug and gene carriers, tissue engineering, absorbable surgical suture, osteogenic and bone filling materials, hemostatic agents, immobilization of therapeutic enzymes, and burn/wound cover dressings [3,4,5,6,7,8]. Collagen is a crucial component of the wound-healing process; it acts as a natural structural scaffold or substrate for new tissue growth and plays an essential role in all phases of wound healing, including hemostasis, inflammation, proliferation, and remodeling [8]. The collagen produced by organisms is defined as endogenous, which consists of three long helicoidally shaped chains of amino acids [2]. Chains of polypeptide constituted by the repeated sequence (Gly-X-Y)_n_ form collagens, where X and Y can be occupied by any amino acid, although these positions are commonly occupied by proline and 4-hydroxyproline (Hyp, Figure 1b) [9,10]. Collagen breaks down due to aging, exposure to ultraviolet light, and tobacco. The degradation of collagen results in wrinkles, sagging skin, stiff joints, and dry skin, and therefore, it is essential to identify new resources of collagen for regenerative tissue applications [11,12].

Exogenous collagen is mostly used in food, biomaterials, and pharmaceutical applications [13]. Due to the wide range of applications of collagen, alternative sources, such as mammalian collagen from cattle and pigs, have been explored. Nevertheless, the outbreak of diseases such as bovine spongiform encephalopathy (BSE) and religious constraints have limited mammalian-based collagen applications [14].

On the other hand, in recent years, the notion of “sustainable development” has become one of the main strongholds in the frame of green economy programs. On a global scale, the urgency to shift towards eco-friendly alternatives for the exploitation of natural resources has been dictated by the need for coping with growing environmental and economic issues. To align with this ecological approach, the European Commission opted for the approval of “Blue Growth”, a long-term strategy meant to support sustainable growth in the marine and maritime sectors. According to Caruso et al. [15], it has been estimated that more than 50% of fish tissues, including fins, heads, skin, and viscera, are discarded as “waste”, exceeding 20 million tonnes of by-products per year. However, the exploitation of seafood by-products represents a growing issue. Moreover, one of the most appealing features characterizing the seafood industry, which has been discarded, is the high content of valuable protein (10–25%) and lipid-rich compounds (17–35%) [16].

Thus, the exploitation of marine by-products as a new source of collagen has attracted increasing attention [17,18] due to their easy extraction, high collagen content, absorption by the human body considering its low molecular weight, biocompatibility, freedom from the risks of animal diseases and pathogens, environmental friendliness, negligible content in biological contaminants and toxins, less significant religious and ethical constraints, and minor regulatory and quality control problems [16]. Consequently, many studies focused their effort toward the extraction and characterization of collagen from skins of different fish, such as small-spotted catshark (*Scyliorhinus canicula*), rabbitfish (*Chimaera monstruosa*), lantern shark (*Etmopterus* spp.), catshark (*Galeus* spp.), cuckoo ray (*Leucoraja naevus*), common Atlantic grenadier (*Nezumia aequalis*), cod (*Gadus morhua*), and the scales and fins of *Catla catla* and *Cirrhinus mrigala* [14,19,20,21].

Collagen from fish skin, bones, and fins have a low denaturation temperature (25–30 °C for most fish species) compared to mammalian collagen (39–40 °C) and variable composition, which limits its use in biomedical applications [22,23]. The low amino acid content (proline and Hyp) in marine collagen causes the low denaturation temperature, which makes fish collagen challenging to handle as it denatures at human body temperature. At present, collagen from marine invertebrate organisms is under investigation, including jellyfish and marine sponges [24,25,26].

The principal roles of collagen fibers in the tissues of vertebrates are to prevent premature mechanical malfunction and assist in storing, dissipating, and transmitting energy from the musculoskeletal or externally applied forces. Collagen fibers provide structural support to all body organs and ensure the firmness, elasticity, and strength that is needed for effective locomotion, tissue regeneration, and repair through the mechanochemical transduction processes [25]. Collagen takes part in the construction of a fibrous network of cells called fibroblasts, which form the base for the growth of new cells. In areas such as the dermis, collagen plays an active role in the protection of the skin by inhibiting the absorption and spreading of pathogenic substances, environmental toxins, micro-organisms, and cancerous cells [2,26]

There are several reviews on collagen resources for the development of biomaterials [9,27], hydrolyzed collagen [28], and sources and applications of marine collagen [10,29]. This review article aims to present an updated overview of the status of collagen isolation from fish by-products with a particular focus on the parameters involves in the isolation of collagen from fish by-products using papers published from January 2014 to June 2020.

## 2. Collagen Formation, Stability, and Molecular Structure

In vertebrates, such as mammals, fish, and birds, 28 different types of collagen, which are coded by at least 45 different genes, can be found [30]. Collagen is the main structural protein found in the extracellular matrix (ECM), making up around 25% to 35% of the body’s total protein content. There are at least 28 types of collagen depending on the domain structure and their superstructural organization, but 80–90% of the collagen in the body consists of types I, II, and III. The most abundant type of collagen in the body, type I, can be found in the bones, skin, tendons, and organs. Type II can be found in cartilage, and type III can be found in reticular fibers, blood, and skin. Collagen type III can be found in the skin, vessel walls, and reticular fibers of the lungs, liver, and spleen (Figure 1). Types IX, XIV, and XIX (FACIT: fibril associated collagens with interrupted triple helices) are associated in low amounts with the fibril-forming types [30] and types IV, XIX, and XVIII are found in basement membranes of cell membranes. Types I to IV collagens are the most prevalent invertebrates.

A single collagen molecule is approximately 300 nm long and 1.5–2 nm in diameter and multi-collagen molecules make up larger collagen aggregates, such as fibrils [31]. Collagen is characterized by a triple-helical α-domain(s) structure named “tropocollagen”, as it consists of three distinct α-chains [31]. These are three parallel polypeptide strands, each with a conformation of a left-handed helix coiled around each other in a rope-like manner to form a right-handed triple helix or “superhelix”, which is the overall tightly packed triple-helical form of the molecule stabilized by many hydrogen bonds [2,30]. The coiling of the three left-handed helices into the right-handed triple helix requires that every third amino acid is a glycine (Gly) residue, while many of the remaining positions in the chain are filled by proline and Hyp [2,31]. Gly is required in the third position because at the crowded center of the three-stranded helix, there is no space for a larger side group than Gly’s single H-atom. The sequence is a repeating pattern of X-Y-Gly, where X and Y may be attributed to any other amino acid residues. However, the X and Y positions are often occupied by proline and Hyp, respectively. The form Pro–Hyp–Gly is the most common tripeptide (10.5%) in collagen [2].

Due to the unique amino acid sequence, the tight collagen triple helix is particularly stable, and having Hyp in the X position is thought to contribute to the stability of the helix. The hydroxyl group of Hyp plays an essential influence on stabilizing the triple helix due to bonding with the pyrrolidine ring. It increases the denaturation temperature and denaturation enthalpy of collagen. Moreover, a network of water molecules surrounds the triple helices, ensuring the thermal stability of the triple helices due to the high denaturation enthalpy, which directly reflects the number of hydrogen bonds, as well as functions as a sensitive parameter of the degree of the triple helical structure [30]. In contrast, the denaturation temperature reflects an entropic and an enthalpic contribution to the stability of the collagen triple helix. Interchain hydrogen bonding stabilizes the three α-chains in the helix, and it renders the molecule moderately resistant to attack by other molecules [31]. These hydrogen bonds, which occur when the amino group (NH) of a Gly residue forms a peptide bond with the carboxyl groups in an adjacent polypeptide, help to hold the three chains together [32].

### 2.1. Marine Collagen

**Figure 2:**
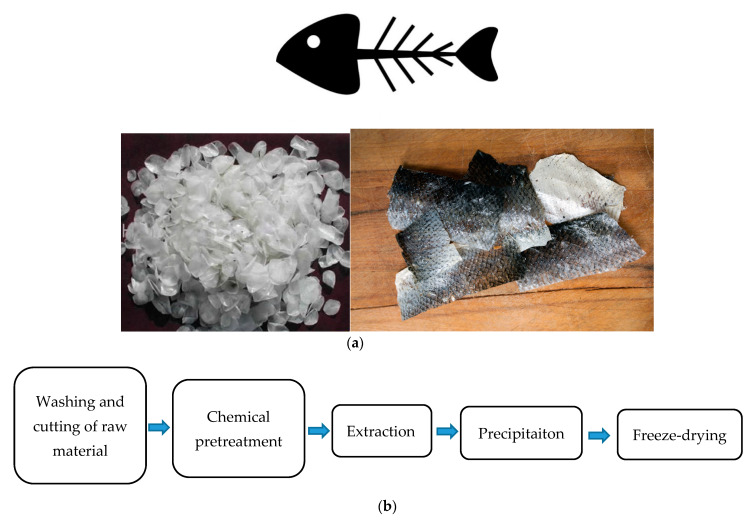
(**a**) Fish bone, scales, and skin as sources of collagen. (**b**). Collagen extraction procedure from fish by-products.

Marine collagens such as fish skin, bone, cartilage, and scales, including both marine vertebrates and invertebrates sources, are more bioavailable compared to bovine or porcine collagen and have a higher absorption capability (up to 1.5 times more efficiently into the body) [33] and more rapid bloodstream circulation due to their low molecular weight and small particle size [34]. In addition, marine-based collagens are similar to conventional bovine and porcine collagen in terms of amino acid composition and biocompatibility [35]. Fish collagen can be obtained from various fish by-products such as fishbone, scales, and skins (Figure 2a) that are consumed daily in different parts of the world and result in a large amount of waste—50% to 70% of original raw materials, which is generated from fish shops and processing factories [36].

#### 2.1.1. Fish Skin

Fish skin typically contains type I collagen with a high degree of purity (around 70%) depending on the species age and season [37]. Collagen from fish skin demonstrates an excellent capacity to retain water (about 6% of its weight in exposure to 63% humidity for 24 h) and exhibits no irritant potential, thus being suitable for dermal applications [38]. A study by Blanco et al. [37] on collagen from the skin of two species of teleost and two species of Chondrichthyes showed a collagen denaturation temperature in the range of 23 to 33 °C while collagen isolated from codfish skin denatured around 16 °C, which possibly could be due to the habitat of the species [39]. However, collagen from the skin of tilapia, catfish, pomfret, and mackerel requires a low extraction temperature (slightly lower than 13.26 °C), long extraction time (74 h), and generates yields of 2.27% (based on dry mass content) [40]. In other work, Wijaya et al. [41] investigated the allergenic properties of collagen from Parang-Parang fish skin. Isolation of the collagen was done by using 0.1 M NaOH in 12 h, and it was hydrolyzed using 0.5 M acetic acid (AcOH) before the experiment. Non-collagen protein content was 0.2163 mg/mL, with a 1.915% yield. Govindharaj et al. [42] investigated the utilization of eel skin-derived collagen (type I) for 3D printing applications. The final yield of the collagen was around 4.2%. A similar result has been reported in which the yield of the collagen extracted from eel fish was 4.7% [43]. Ahmed et al. [44] investigated using bacterial collagenolytic proteases (CP) to extract collagen from fish skin as an alternative method. They used two bacteria, *Bacillus cereus* FORC005 and *Bacillus cereus* FRCY9-2, to produce CP. The total yield of collagen by the bacteria treatments and combined with acid-soluble collagen was 188 and 177 g/kg, respectively, which were greater than the yield from acid extraction alone (134.5 g/kg). Another approach to extract collagen from fish skin is the utilization of water acidified with CO_2_ that was used to isolate collagen from Atlantic cod (*Gadus morhua*) [45]. Acidified water-extracted collagen showed a total content of proline-like amino acids of 151/1000 residues, with an extraction yield of 13.8% (*w/w*) [45].

#### 2.1.2. Fish Scale

Fish scales constitute a considerable amount of waste from the fish processing industries. Recent studies [46,47,48] suggested that collagen obtained from fish scales possess properties typical of type I collagen consisting of two α1 chains and one α2 chain. A study on collagen from the scales of tilapia (*Oreochromis* sp.) showed a high denaturation temperature (57.9–79.0 °C) that was possibly due to its high amino acid content and higher intra/interchain bonds (hydrogen bonds, dipole–dipole bonds, ionic bonds, and Van der Waals interactions) [47]. In contrast, collagen isolated from fresh carp (*Cyprinus carpio*) scales showed a low denaturation temperature (32 °C) [48]. Collagen from the scale of tilapia, catfish, pomfret, and mackerel requires a higher extraction temperature (range 16.6–19.03 °C) and longer extraction time (77.51 h), with lower extraction yields (0.13%) compared to fish skin (4.3%) [49]. Collagen from fish scales has also shown to have proper water absorption (13.3%) and retention properties (15%), which make it suitable for medical and therapeutic applications [47]. Collagen-based wound dressing (paste, and sheet) from the scales of tilapia and grey mullet has shown excellent antimicrobial activity *Staphylococcus aureus* and *E. coli* through a disk diffusion method. Moreover, the wound dressing exhibited high wound closure capacity (up to 99.63%), indicating the role of fish scale collagen in the acceleration of re-epithelialization [46,50]. However, due to a high amount of calcium (16–59% mineral content in weight) in fish scales, decalcification is required to be performed by ethylenediaminetetraacetic acid (EDTA) [51].

#### 2.1.3. Fish Bones

Collagen from fishbone shows properties of type I collagen consisting of two α1 and one α2 chain [52,53]. A study on collagen from the bones of Tilapia (*Oreochromis mossambicus*) shows a denaturation temperature of 32.5 °C [54]. Collagen from fish bones of tilapia, catfish, pomfret, and mackerel requires a high extraction temperature (16.6–19.03 °C) and shorter extraction duration (73.16 h) compared to fish scales and skin, and it has lower extraction yields (0.64%) compared to fish skin [49]. Ramli et al. [55] have reported that the collagen from *Lutjanus* sp. bone with a yield of 4.535% (with a protein concentration of 8.815 mg/mL) can be used as a natural anticancer agent. High-intensity pulsed electric fields (PEF) is one of the main approaches to extract collagen from fishbone [56]. In a study by He and co-workers [57] a combined extraction method of semi-bionic extraction (SBE) and PEF treatments were applied for the isolation of calcium, chondroitin, and collagen from waste fish bones. In the SBE method, the digestion and absorption process of the human gastrointestinal tract is simulated through a repetitive acid and alkaline extraction and different pH ranges. Utilizing PEF of 22.79 kV/cm, the authors extracted 3.87 mg/mL of collagen and the combined technique of PEF and SBE was reported as efficient for the isolation of collagen, calcium, and chondroitin from fishbone [56].

The same group [58] in a recent study achieved a maximum collagen yield of 16.13 mg/mL from fish bones using pepsin 1%, with a PEF strength of 20 kV/cm. Desalting is also suggested as an essential process in bone collagen extraction due to its high hydroxyapatite and calcium content, which are removed by EDTA or HCl during the pretreatment; however, using HCl can degrade the collagen [54].

#### 2.1.4. Fish Cartilage

Collagen from fish cartilage consists predominantly of type II collagen, and some other types of collagen in minor quantities such as type IX and type XI found in the nasal cartilage of Hoki (*Macruronus novaezelandiae*) [38]. Type I collagen is found in the cartilage of Sphyrna lewini, Dasyatis akjei, and Raja porosa [59]. In Amur sturgeon (*Acipenser schrenckii*) cartilage, type I collagen was found in acid-solubilized and salt-solubilized collagen and type II with other minor types was found in pepsin-solubilized collagen [26]. Hoki cartilage-derived collagens have been reported to have similar α chain assembly, amino acid composition, and structure to collagens in mammalian cartilage; and they could have potential in biomaterials for the treatment of cartilage-related diseases [60]. The physicochemical and antioxidant properties of collagen isolated from silvertip shark (*Carcharhinus albimarginatus*) were evaluated by Jeevithan et al. [47]. Type II acid-soluble collagen (ASC), pepsin-solubilized collagen (PSC), and type II gelatin were extracted from this cartilage. The denaturation temperature of type II gelatin was 32.5 °C, which was higher than that of two other collagens (PSC and type II gelatin). However, the antioxidant activity against 1,1-diphenyl-2-picrylhydrazyl radicals and the reducing power of PSC was higher than that of type II ASC and type II gelatin. The collagens isolated from the silvertip shark can be a suitable candidate for biomedical applications due to its higher antioxidant activity. In another study, Luo et al. [61] obtained ASC and PSC from cartilages of Siberian sturgeon (*Acipenser baerii*) with yields of 27.13 ± 1.15% and 14.69 ± 0.85%, respectively. According to the results, collagens from this cartilage might be the suitable alternatives of mammal type II collagens.

Fish cartilage collagen shows a lower denaturation temperature than bovine collagen, in the range 26.3 °C to 35.9 °C, which is attributed to the habitat of the species; for example, hoki collagen’s low denaturation temperature is consistent with a cold-water habitat [59,62,63]. Alternatively, collagen from chum salmon (*Oncorhynchus keta*) denatures at 19 °C, while such shark collagen denatures around 30 °C [35]. This temperature instability limits the application of some collagen-derived biomaterials in human medical applications. Therefore, further investigation is required to find an equally sustainable alternative to fish collagen with a higher denaturation temperature, which is more convenient for biomedical applications and could guarantee better performance in terms of thermal and mechanical stability. In addition to thermal stability, the composition/structure of collagen should be similar to mammal collagens as much as possible, particularly for biomedical applications.

Table 1 shows a summary of recent studies on the isolation of some marine-derived collagens.

## 3. Collagen Extraction Methods

Different extraction methods can be performed based on the marine sources. However, the general procedure of collagen isolation includes preparation, extraction, and recovery (Figure 2b).

The preparation mostly consists of washing, cleaning, the separation of animal parts, and size reduction by cutting or mincing the samples for facilitating the following pretreatment of the samples [70]. After the preparation, a mild chemical pretreatment is performed to increase the efficacy of the extraction and remove non-collagenous substances. Generally, depending on the raw materials and the extraction method, different pretreatments can be performed (alkaline or acid treatment). Pretreatment is used with a diluted acid or base to break down the crosslinked collagen before the extraction because of crosslinked collagen in the connective tissue of animals [71]. Indeed, partial hydrolysis takes place, which keeps the collagen chains intact [72].

In the acidic form of pretreatment, the raw materials are immersed in the acid solution. The penetrated solution in the collagen structure allows it to swell two or three times its initial volume, which leads to the cleavage of the non-covalent inter and intra-molecular bonds [73]. The alkaline pretreatment is mostly performed by using sodium hydroxide (NaOH) and calcium hydroxide (Ca(OH)₂) for a period that can take from a few days to several weeks [72]. However, using NaOH is more convenient due to the higher swelling ability leading to facilitating the extraction of collagen by increasing the transfer rate of the mass in the tissue matrix [74].

Moreover, prior to the extraction phase, the demineralization of the raw materials is required to enhance collagen extraction efficiency from the part of the body with a high amount of minerals such as bone, cartilage, and scales. Usually, demineralization can be done by using either EDTA or HCl [75,76].

Collagen fibers exist as a triple helix, with stable inter and intra-molecular hydrogen bonds crosslinks, which make the collagen fibers insoluble in water. Therefore, for the extraction, the use of specific extraction techniques is required to increase the solubilization of collagen proteins and accomplish their isolation. Extraction of acid-solubilized collagen (ASC), extraction of pepsin-solubilized collagen (PSC), deep eutectic solvent (DES), and supercritical fluid (SF) extractions are the major methods reported in the literature [69,77] for collagen isolation from fish by-products.

### 3.1. Acid Extraction Procedure

ASC is known when collagen is extracted by only acid. Acids (such as HCl and AcOH) hydrolyze the triple helix of collagen and solubilize its single chains in solution, where the depolymerization of heavyweight proteins into shorter peptides (0.3–8 kDa) takes place [77]. The interaction between the acid and the collagen molecules breaks the crosslinks present in the collagen helix and increases the extraction efficiency. Therefore, it is of great interest to investigate the extraction efficiency using different acids to maximize the purity and yield of the collagen extracted. The cleavage of the triple helix carried out by acids is schematically shown in Figure 4.

AcOH is one of the most common compounds through which collagen extraction from animal and marine sources is carried out [19]. The range of concentrations for the acid extraction solution is between 0.5 and 1 M, which allows the cleavage of intra and inter-molecular crosslinks without affecting the structure of the collagen chains. Although in most studies, researchers use 0.1 or 0.5 M AcOH to extract collagen from fish skin, Tan et al. [78] investigated the effect of using different acids (AcOH, hydrochloric acid, citric acid, and lactic acid, W:V = 1:50), liquid-to-solid ratios, and various pH (1.8, 2.1, 2.4, 2.7 and 3.0) on the extraction of collagen from catfish skin by different methods (acid, homogenization-aided, and pepsin-aided extraction methods). The pepsin and homogenization-aided (PHSC) method exhibited the highest protein recovery (64.19% at pH 2.4 by HCl). In terms of acid extraction method, the rate of the protein recovery isolated from the skin by HCl at pH 2.4 was 42.36% (the highest) followed by extraction with pH 2.7 AcOH (39.45%). This study conflicted with other studies that reported the lower collagen extraction yield by HCl compared to AcOH. The reason might be due to the different concentrations of acid that have been used, and the pH of the mixture was not maintained at the beginning pH, which tended to change over the time of extraction.

The effect of AcOH with a range of 0.2–1.0 M on collagen extraction from sole fish skin was determined by Arumugam et al. [79], while the other variables were constant. The yield of collagen increased gradually with increasing of the AcOH concentration so that a maximum yield was 15.968 mg/g at 0.6 M of AcOH. However, beyond 0.6 M, the collagen yield reduced.

Yang et al. studied the interaction between collagen molecules and the AcOH solution. The aggregated state of collagen molecules and AcOH concentration were associated with each other so that the collagen critical aggregation concentration increased from 0.518 to 1.581 mg/mL for alteration from 0.1 to 2.0 M of AcOH concentrations. The rheological behavior of the collagen solution as a function of AcOH concentration was investigated to understand the interaction between collagen molecules and the acidic solvent [80]. All samples exhibited shear-thinning behavior under steady shear tests, which can be used as 3D printing bioinks. With increasing concentration of AcOH, the ability of collagen to flow was increased, which could be due to a decrease of viscosity. In addition to AcOH, citric acid and lactic acid have been used for collagen extraction [78]. Table 2 summarizes the results obtained in experiments that employed AcOH as an extraction medium.

### 3.2. Pepsin-Aided AcOH Extraction Procedure

**Figure 3:**
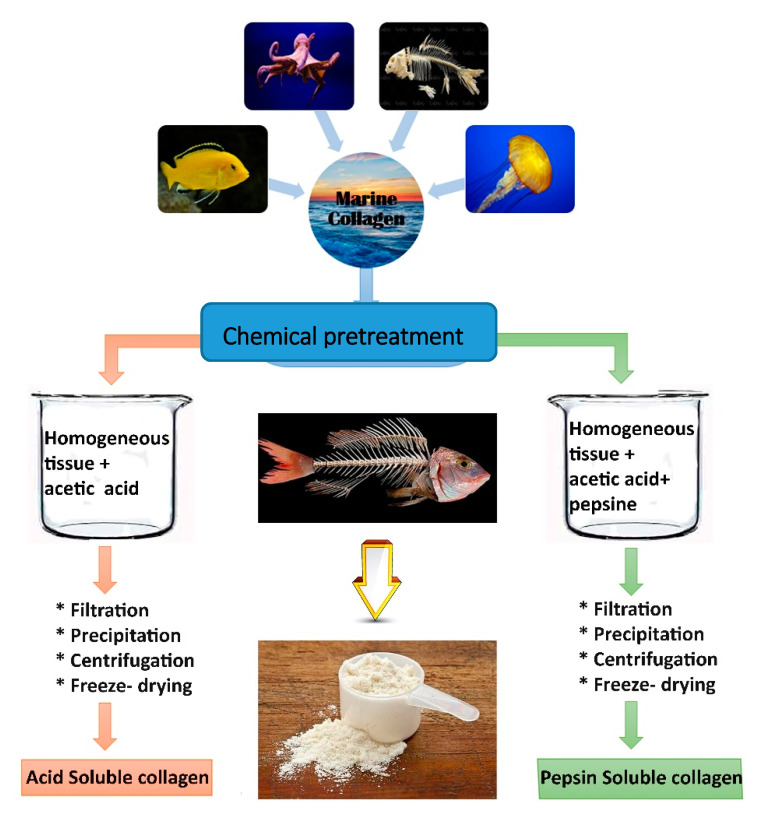
Difference between acid-soluble collagen (ASC) and pepsin soluble collagen (PSC) extraction methods.

The pepsin-aided AcOH extraction is the second primary method for collagen extraction, which allows cleaving of the telopeptide regions of the triple helix, facilitating the leaching of collagen peptides in solution and increasing the extraction yields. PSC is a known pepsin that is added to the extraction process (Figure 3) [64]. Therefore, many studies [19,86] have employed an enzymatic pretreatment, using pepsin to digest the telopeptide ends of the collagen chains to facilitate the removal of proteins from the remaining matrix. Several factors play a critical role in the efficacy of pepsin-aided AcOH extraction methods such as pepsin concentration, hydrolysis time, and solid–liquid (S/L) ratio on pepsin-solubilized collagen that need to be optimized for more valuable results [87]. The effect of pepsin concentration on the extraction yield of pepsin-soluble collagen was investigated when the other extraction parameters were constant. Increasing the pepsin concentration (800 to 1200 U/g) caused an impressive increase in the isolation yield (66.35% to 79.93).

Moreover, the influence of the concentration of both AcOH and pepsin enzyme in collagen isolation from nilem fish skin was investigated by Junianto et al. [88]. For this aim, they used three different concentrations of AcOH and pepsin enzyme. Results showed that the maximum yield of collagen extraction (6.18%) was obtained at the combination treatment concentration of 0.7 M solution of AcOH by the pepsin enzyme at 1.0%. Table 3 summarizes the extraction conditions and the PSC yields obtained in experiments involving the isolation of collagen from different marine species.

## 4. Influence of Extraction Parameters on Collagen Yield

There are several parameters, such as temperature, time, and solvent concentration, which affect the extraction yield of collagen from fish sources. The effect of each of these parameters should be considered and optimized to define the suitable ranges to perform the collagen isolation experiments.

### 4.1. Effect of the Temperature on Collagen Extraction

The collagen extraction temperature depends on the type of substrates for collagen extraction. As shown in Table 2 and Table 3, the temperature range for collagen extraction from fish skin is generally between 4 and 25 °C, since fish collagen has a denaturation temperature between 30 and 40 °C.

Furthermore, when using pepsin, it is more prudent to keep temperature low (4–10 °C), since this enzyme is highly sensitive to high temperatures (above 60 °C), which could lead to its self-digestion and deactivation. When the cleaving action of pepsin is over, the samples are generally heated to 90 °C for a few minutes to deactivate the enzyme, preventing it from further degrading the collagen structures [65].

Concerning the mass transfer taking place during extraction, an increase in temperature will induce a drop in the viscosity of the extraction solution, thus resulting in the rise of the overall mass transfer rate. Nevertheless, increasing the temperature above the denaturation point of collagen would lead to thermal degradation of the isolated proteins. Collagen extraction from fish sources is mainly carried out between 4 and 10 °C, which allows pepsin to cleave the crosslinks in the collagen triple helix without damaging the structure of the peptides.

### 4.2. Effect of the Extraction Time

The effect of extraction time on collagen yield could be explained in terms of the mass transfer rate. Extraction is strongly controlled by the diffusion process, which is time-dependent; therefore, the recovery of collagen will keep increasing with the extension of the extraction time.

Nevertheless, excessive extraction durations could lead to the degradation of the leached peptides. In such a case, the acid solution starts breaking down the collagen chains, provoking their decomposition and decreasing the final extraction yield. Furthermore, long extraction times would make the extraction process unsuitable for industrial scale-up. For example, Arumugam et al. [79] reported that the yield of sole fish skin collagen was increased with the increment of time, but then the yield was decreased. The optimum time for achieving a maximum yield was 36 h. However, in another study, increasing the extraction time altered the yield from 15.3 to 19.3% [96].

Furthermore, Alfaro et al. [97] investigated the optimum condition for extracting collagen from Wami tilapia. The effect of four parameters, such as NaOH concentration, H_2_SO_4_ concentration, and extraction temperature, as well as extraction time, were evaluated. The isolation time effect was studied at various times (3–15 h), and the results showed which time has a significant influence so that increasing the extraction time from 3 to 15 h resulted in an increase of 1.72% in the yield [97].

### 4.3. Effect of Solvent Concentration

Most of the AcOH collagen extraction methods were carried out using 0.5 M of AcOH (Table 2). Higher molarities would cause degradation of the peptides, thus decreasing both the yield and purity of the final product [79]. Arumugam et al. [79] studied the effect of AcOH concentration on the collagen yield of sole fish skin using a range of concentrations between 0.2 and 1 M while all the other experimental variables were kept constant. As the AcOH concentration reached 0.6 M, the yield of collagen increased to 16 mg of collagen/g of fish skin. Beyond 0.6 M, the collagen yield dropped to 12.5 mg of collagen/g of fish skin, because of the degradation effect induced by the excess of acid.

Moreover, other organic acids, such as citric acid and lactic acid, can be used for collagen extraction. Inorganic acids (e.g., HCl) can also be applied to extract collagen; however, their efficiencies are lower compared to the organic acids. Organic acids are more effective in the solubilizing of non-crosslinked collagen and breaking of some of the inter-strand crosslinks in collagen [74]. Therefore, due to the low cost and excellent performance, AcOH is the most used organic solvent for collagen isolation from fish by-products.

Another critical factor concerning AcOH extraction is the effect of the pepsin used to cut the telopeptide ends of the collagen triple helix. The influence of pepsin concentration (between 1 and 10% [67,91]) must not be neglected: more enzyme molecules increase the digestion rate of the telopeptide ends, thus speeding up leaching and guaranteeing a more efficient extraction of the collagen peptides. When pepsin concentration reaches a threshold value (about 10% [91]), which depends on the nature and amount of starting material used, all the telopeptide regions undergo cleavage. Then, more pepsin above the threshold could decrease the extraction yield, since it would start degrading the solubilized collagen molecules.

### 4.4. Effect of Solid-to-Liquid Ratio

The S/L ratio is defined as the amount of solid collagen source divided by the mass of liquid solution employed for the extraction. In general, increasing the amount of solution enhances the interactions between the free protons and the amino acids of the collagen chains, thus improving the cleavage of the crosslinks present in the collagen helix. The decrease of the solid-to-liquid ratio enhances the depolymerization rate of the peptides, since a large amount of acid leads to fragmentation of the collagen chains, thus resulting in a product rich in lower molecular weight peptides [79]. Since the molecular size of these proteins strongly determines their functionality in biological processes, the solid-to-liquid ratio needs to be tuned regarding the application of the isolated product.

**Figure 4:**
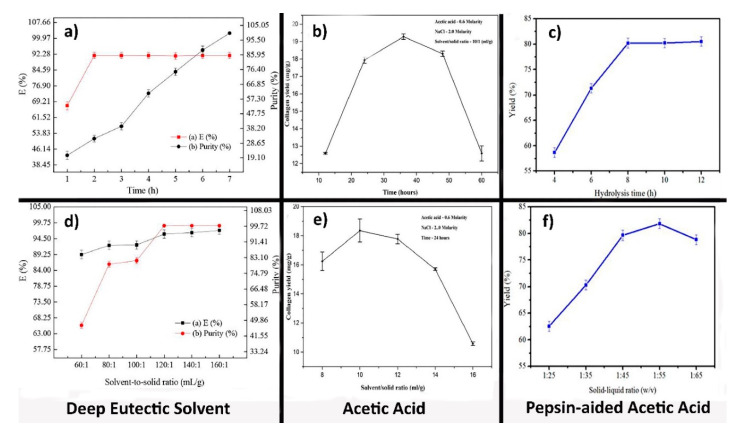
Effect of time and solid/liquid ratio on the yield of collagen from (**a**,**d**) cod skin [77] Copyright (2017) American Chemical Society. (**b**,**e**) sole fish skin, reprinted from with permission from Elsevier (4851440916595) [79], and (**c**,**f**) giant croaker skin, reprinted with permission from [87] (open access Creative Common CC BY license.)

As shown in Table 2 and Table 3, some studies [64,74] employed an S/L ratio between 1/40 and 1/50, which is lower compared to other studies [79,81] where a solid-to-liquid ratio of 1/10 was used. As the amount of solvent is increased, the fish tissue will be exposed to a larger quantity of fresh solution, thus boosting the solubilization rate of collagen peptides. The different S/L ratios such as 1/25, 1/35, 1/45, 1/55, and 1/65 were used to study the effect of S/L ratio on the extraction yield of pepsin-soluble collagen from the skin of giant croaker (Nibea japonica) when the pepsin concentration was 1200 U/g and the hydrolysis time was 8 h in 0.5 M AcOH by Yu et al. (Figure 4) [87]. The yield of extraction was significantly increased with an increasing S/L ratio from 1/25 to 1/55 so that the 1/55 ratio was selected as the optimum condition. Then, with increasing the S/L ratio from 1/55 to 1/65, the extraction yield was decreased. The optimal solid-to-liquid ratio range for AcOH extractions is reported to be between 1/40 [74] and 1/60 [83], approximately.

## 5. Other Extraction Methods

Although acid extractions methods are the main approaches for collagen isolation, high acidity, long processing time, and high temperature in acid extraction methods can negatively induce a high degradation of soluble collagen chains. Therefore, new processing technologies that allow better preservation of the target peptides need to be investigated. Hence, many researchers focused their attention on the development of innovative and sustainable extraction techniques to reach higher collagen yields employing cheap and less toxic materials.

### 5.1. Deep Eutectic Solvent (DES) Extraction

DES is a mixture of two compounds: one acts as a hydrogen bond acceptor (HBA), while its partner acts as a hydrogen bond donor (HBD). The DES method is mostly based on abundant, low-toxic, and biodegradable natural components (choline chloride, oxalic acid, urea, ethylene glycol), which make DES particularly suitable for the extraction of valuable chemicals from animal, marine, and plant by-products [98,99].

Out of the six different DES investigated by Bai et al. (2017) [77], the mixture of choline chloride (CC) and oxalic acid (OA) has proved to be the most effective one, showing extraction efficiencies close to 90% for cod skins. This value is almost four times higher than the extraction yields obtained with AcOH extractions from the skin of tilapia (27.2%) [84]. Therefore, the CC-OA co-solvent represents a sustainable alternative for the isolation of collagen peptides from marine by-products.

The effect of the CC/OA ratio in the DES extraction method significantly influences the extraction efficiency of collagen. Various CC/OA ratios were tested in the range between 1/0.6 and 1/1.4, maintaining the systems analyzed at the same temperature (i.e., 45 °C). By increasing the quantity of oxalic acid with a constant amount of choline chloride, at first, a raise in the extraction efficiency was observed until a ratio of 1/1 is reached; then, a slight drop in the yield was measured. As the amount of oxalic acid is increased, the number of free protons in solution grows as well, thus inducing a rise in the number of interactions with the collagen helices and facilitating the leaching of peptides. Nevertheless, oxalic acid also increases the viscosity of the solution until the rate of mass transfer is negatively affected.

Furthermore, Bai et al. [77] investigated the effect of CC-OA extraction temperature on the collagen yield of cod skins. The efficiency of collagen extraction increases steadily with temperature until reaching a plateau from 65 to 75 °C. Higher temperatures induce a decrease in the viscosity of the solution, which increases the mass transfer coefficient and, therefore, the leaching rate. In addition, increasing the temperature improved the purity of obtained collagen until a minimum point is reached corresponding to 55 °C. Furthermore, due to the enhanced polymerization of the collagen chains, above 55 °C, the efficiency raises to 95.68%.

The effect of extraction time and S/L ratio on collagen yield in three approaches of isolation, including DES, acid, and pepsin-aided AcOH from cod skin, sole fish skin, and giant croaker skin, have been compared (Figure 4a–f). The yield of collagen for pepsin-aided AcOH extraction procedures and DES extraction was higher (around 85% at 1/55 S/L ratio) than acid extraction procedures. However, the highest performance belongs to DES extraction (99.72%). Although the collagen yield at first increased and then decreased in the acid isolation method over time, in two other approaches, this amount increased early and afterward was constant or increased with a low slope [77,79,87].

### 5.2. Supercritical Fluid Extraction (SFE)

Supercritical fluid extraction (SFE) has become one of the most popular green extraction techniques to extract chemical compounds. Several advantages have been reported for supercritical fluid extraction in comparison with traditional or classical extraction processes, such as improved selectivity, higher extraction yields, better fractionation capabilities, and lower environmental impact [69]. SFE is based on the use of fluid at pressures and temperatures beyond the critical point to achieve significant physical changes that will modify its capabilities as a solvent. CO_2_ is the most commonly used molecule for the SFE method due to low toxicity, cost-effectivity, high availability, stability, flammability, environment acceptability, and mild operating condition (moderate pressure and temperature). Furthermore, CO_2_ could be released after extraction from aqueous media, and therefore, a purified compound will be obtained [45,100,101]. Recently, SFE has been used for collagen extraction from marine waste instead of traditional acid-based extraction.

For cod skin, the yield of collagen has been reported to be 13.8, 5.72, and 11.14%, in which these values were obtained with SFE, acid, and pepsin-aided AcOH extraction procedures, respectively [45,91]. In another work, SFE has been used to isolate collagen and gelatin from marine sponges. For the achievement of the highest yield, the operation conditions were optimized so that the yield of collagen was approximately 10% [102].

### 5.3. Extrusion and Ultrasound-Assisted Extraction of Collagen

High-temperature short-time (HTST) and high shear force processes are two characteristics of extrusion cooking that have been used for many years to produce human food and animal feed. Forming and expanding cereals, cooking in the food industry, and texturizing proteins can be performed by the extrusion cooking method. The extrusion process is followed by various reactions such as thermal treatment, protein denaturation, grinding, hydration, gelatinization, shearing, mixing, shaping, expanding, partial dehydration, texture alteration, and microorganisms destruction or other toxic compounds [103,104]. Generally, some of the essential advantages of extrusion cooking are easy operation, continuous production, little required labor, low labor cost, and limited waste, as well as a multiplicity of products. As a result, the extrusion method can be used for the pretreatment to extract collagen from fish by-products. Extrusion-hydro-extraction (EHE) aims at increasing the solubility of the collagen chains present in the sample using an extrusion pretreatment. Huang et al. [90] reported that the high pressures reached in tilapia scales during extrusion allow yields of collagen between 7.5 and 12.3%, which is 2–3 times higher than yields of a normal AcOH extraction procedure.

However, other studies focused on using a sonication pretreatment to increase the extractability of the collagen peptides. Ultrasounds induce cavitation in the liquid solvent, forming microbubbles whose collapse damages the fish tissues and increases the contact area between liquid and solid with a range of wave 20 to 1000 kHz. Shear forces produced by cavitation bubbles relate with their size so that with an increase in the ultrasonic frequency, the size of the bubbles decreases, and the shear forces increase [105,106,107,108]. Zou et al. [105] reported that the yield of the collagen extracted from soft-shelled turtle calipash increased by 16.3% when using ultrasound pretreatment (24 min, 200 W, and 24 kHz sonication) compared to the yields measured in conventional AcOH extractions. Therefore, both techniques impact the final collagen yield positively and should be investigated in future research.

## 6. Collagen Characterization Methods

Generally, good knowledge and characterization of the collagen extracted from marine species will lead to establishing a logical relationship between results and the features of the structure. It can help researchers control results by changing the structure [109]. Several techniques can be used to characterize marine collagen (in solution or solid-state), addressing structural, chemical, and morphological properties.

### 6.1. Chemical Composition of Collagen

Fourier transform infrared (FTIR) is a tool that is used to evaluate and recognize the collagen presence and chemical composition, and it is also used to identify its type. Moreover, it can be used to compare the collagen composition extracted with different approaches or investigate the effect of isolation methods on collagen composition [13,47,82]. For example, Chuaychan et al. [82] studied FTIR spectra for collagens extracted by using acid and pepsin-aided AcOH extraction procedures from scales of seabass. The FTIR spectra suggested that the collagen analyzed corresponds to type I and indicated that the functional groups present in the triple helix were not damaged by the treatments employed. Thus, the functional groups were not affected by the extraction process. Nevertheless, it is possible to notice differences in the width and height of the corresponding signals in several graphs, which can be due to the different experimental conditions of each study (such as different solvents with different concentrations) [13,47,82]. As can be seen in Table 1, almost all of the collagen extracted from marine species was type I. Table 4 shows the main functional groups of collagen type I identified by FTIR.

### 6.2. Characterized Purity of Collagen and Breakdown

Electrophoresis, such as sodium dodecyl sulfate-polyacrylamide gel electrophoresis (SDS-PAGE), is typically employed to identify protein patterns and determine the molecular weight distribution of the collagen peptides. By using this tool, it is possible to separate protein and their fragments from each other based on their size. Although the protein with a larger chain is trapped in the gel, the smallest fragments travel through the gel net [10]. Determination of the collagen type by comparing collagens obtained from other sources as a reference is possible when the collagen bands are similar. In addition, the identification of amino acid sequences in the same type of collagen is feasible [10,110]. Table 5 displays the characteristics bands that are considered as the gold standard for the identification of collagen type I peptides. Collagen comprised of three alpha chains can be either the same or different alpha chains depending on the type of collagen. For example, type I collagen consists of two alpha 1 chains and one alpha 2 chain, while type II collagen contains three alpha 1 chains [111]. Moreover, dimers (β-chains) or trimers (γ-chains) can be found in the SDS-PAGE based on their assembly and post-translational modification. Generally, fish collagen consists of two alpha 1 chains and one alpha 2 chain (around 100 kDa) [112].

Moreover, dimers (161 kDa) and trimers have been observed for different discarded fish species (rabbitfish, cuckoo ray, common Atlantic grenadier). Unlike other species, rabbitfish exhibited a very weak peak concerning the beta component, and alpha 2 components are hardly seen.

**Figure 5:**
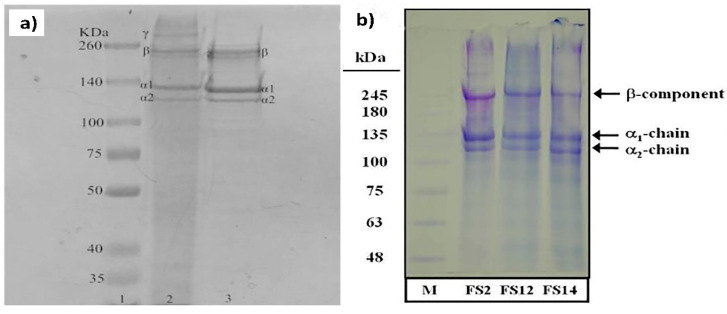
(**a**) Protein pattern for acid-soluble collagen from tilapia skin and scales [84]. Reprinted from with permission from Elsevier (4851441506573), and (**b**) protein pattern for extrusion-hydro-extraction (EHE) procedures from tilapia skin. Reprinted from with permission from Elsevier (4851450130304) [90].

The results of SDS-PAGE collagen extracted from scales of tilapia with two approaches, acid and EHE procedures, are shown in Figure 5. The extraction method employed does not influence the molecular weight distribution of the collagen peptides. In addition, α_1_ band intensity was 2-fold higher than α_2_. This is due to two identical subunits of α_1_ and one of α_2_ in type I collagen [84]. As shown in Figure 5, the molecular weights of the α2 and α1 chains in each case were between ranges of 116–121 kDa and 126–132 kDa, respectively. Instead, the beta band was situated at a higher value (approximately 255 kDa).

### 6.3. Secondary Structure of Collagen

Similar to other proteins, the secondary structure, binding, and folding feature of collagen can be assessed by the circular dichroism (CD), which is based on the measuring of different absorption of right- and left-circularly polarized light [113,114]. Indeed, circular dichroism (CD) can indicate that an extracted collagen is in the native–triple helical structure or denatured form–coil structure [35].

Although circular dichroism is a fast technique, investigation of the folding properties of some proteins such as collagen and collagen fragments takes more time compared to other proteins due to their slow folding process. Hence, a pre-folding should be performed, especially for proteins with unknown folding properties. The protein should be pre-folded for several hours to several days at 25 °C or on ice (in a refrigerator) before conducting circular dichroism (CD) analysis [114]. The supercoiled polyproline secondary structure (type II) of the triple helix of collagen has well-defined CD transitions containing a positive and negative band at 222 and 195 nm, respectively. However, gelatin, as a denaturized product of collagen, does not have any characteristic CD signal, which can demonstrate that the CD signals of collagen are mainly due to the ordered fibril composed of triple-helical units [115].

CD spectroscopy, in combination with other techniques such as rheology, can pave the way for providing useful information about the secondary structure of type-I collagen fibrils and evolution of the collagen fibril signal, resulting in new insights in collagen-based biomaterials.

### 6.4. The Yield of Collagen and Amino Acid Analysis

The yields of collagen extraction calculations for meat and meat products follow the guidelines of an Iso standard (ISO 3496:1994). The ratio of extracted Hyp to its initial concentration in fish by-product is measured as collagen yield to calculate the extraction yield; the amount of Hyp is used because it is an exclusive amino acid for collagen (about 30% of all amino acids in collagen), and its amount is insignificant in other proteins. So, its amount is used to calculate the extraction yield [10]. In Table 2 and Table 3, the extraction yield of collagen from many fish species has been presented.

The separation, identification, and quantification of amino acids were investigated by using chromatographic techniques [116]. Concerning the amino acid analysis, Table 6 shows the amino acid content of collagen samples extracted from several fish species. It is well known that collagen type I is abundant in Gly, alanine, proline, and Hyp [16]. The primary amino acids present in the extracted collagens are alanine, Gly, proline, and Hyp, thus confirming the presence of collagen type I. Furthermore, there are no substantial differences between the amino acid profiles in the collagens extracted with the different methods. Therefore, the extraction procedures presented in this review are all suitable for the isolation of collagen type I.

### 6.5. Thermal Properties of Collagen

High temperature causes the structure of collagen molecules to unfold. The maximal transition and denaturation temperatures of collagen are obtained by measuring the flux of calorimetric energy [82]. The thermal properties of collagen are evaluated by differential scanning calorimetry (DSC). With increasing heating in DSC, collagen absorbs heat and, at a specific temperature (different for each species), it starts to unfold [10]. DSC can be used to evaluate collagen thermal stability, which is directly attributed to the presence of amino acids in the collagen chain [82]. For example, collagen with more Hyp content has a higher denaturation temperature due to the hydroxyl group present in Hyp that can participate as a hydrogen donor via α-chains. Chuaychan et al. [82] reported that the collagen extracted with pepsin, which had a higher Hyp content, showed a maximum denaturation temperature of 39.32 °C and a change in enthalpy (ΔH) of 0.91 J/g, while the denaturation temperature and ΔH of the collagen sample isolated with acid was 38.17 °C and 0.72 J/g, respectively. In another study, the Hyp content extracted from the skin and swim bladder of seabass (*Lates calcarifer*) has been reported as 79 and 83 (residues/1000 residues), respectively. The maximum temperature for swim bladder was 35.02 °C, and for skin, it was 33.33 °C. In addition, the ΔH of the swim bladder (0.918 J/g) was higher than the ΔH of skin (0.860 J/g) [118].

## 7. Marine Collagen Biomaterials Application

Due to water solubility, safety, biocompatibility, biodegradability, and easy extractability, as well as low immunogenicity, marine collagen has attracted scientific consideration for biomaterial applications [119]. On the other hand, due to the enormous amount of marine waste by-product such as fish skins, bones, scales, cartilage, and heads, marine-based collagen has been used in various biomaterial applications such as bone tissue engineering, skin tissue engineering and regeneration, cartilage tissue engineering, wound dressing, drug delivery, etc. [15].

One of the main aims of tissue engineering is the regeneration of damaged, diseased, or eliminated organs and tissues using porous, biocompatible, and biodegradable scaffolds [120,121,122,123]. Bone regeneration involves the formation and resorption of bone, which is a complex physiological process [124]. Trauma, infection, skeletal abnormalities, tumor resection, vascular necrosis, atrophic non-unions, and osteoporosis are the agents that cause defects and damage in bones. Allograft implantation, osteoconductive scaffolds, osteoprogenitor cells, free fibula vascularized graft, and distraction osteogenesis are some different strategies to augment the bone regeneration process [27,125]. The osteogenic activity of scaffold fabricated by marine collagen has been studied for bone tissue engineering applications. Elango et al. [126] extracted collagen from blue shark cartilage and then prepared three types of scaffolds of its collagen, including collagen, collagen–chitosan, and collagen–hydroxyapatite. The physical–functional properties, mechanical properties, biocompatibility, and osteogenesis of scaffolds were investigated. The collagen–hydroxyapatite sample had a stiffness of 8.95 MPa, a water bonding of 231%, good biocompatibility, and a low biodegradation rate. To determine the bone differentiation and mineralization of scaffolds, researchers used alkaline phosphatase activity (ALP) after 3 days of incubation of osteoblast cell (hFOB). The ALP of collagen–hydroxyapatite was approximately 35 U/L, which was higher than the other samples’ ALP activity. In other work, the poly (lactic-co-glycolide) nanofibrous membrane was fabricated and further modified by different weight proportions of low-immunogenicity fish collagen (1:100, 5:100, 10:100, and 15:100) and nanohydroxyapatite (5:100, 15:100, and 25:100). The tensile strength of the nanofiber sample due to the interaction between fish collagen and poly (lactic-co-glycolide) significantly improved so that the tensile strength of the 5:100 sample was 6.5 MPa, which was six times higher than the tensile strength of the sample based on poly (lactic-co-glycolide). Moreover, the fish collagen accelerated the biodegradability so that the mass remaining of the nanofiber samples with fish collagen after 70 days was 75%, while the poly (lactic-co-glycolide) nanofibrous remaining mass was 90%. The results illustrated that the samples with weight proportions of fish collagen and nanohydroxyapatite of 5:100 and 15:100 respectively had the highest ALP activity [127].

Ascartilage does not have the ability to self-regenerate, cartilage tissue engineering attempts to repair or regenerate injured or diseased articular cartilage. Pugliano et al. [128] showed that the jellyfish collagen implant could be used to develop cartilage regeneration with therapeutic molecules. Moreover, Mredha et al. [129] have successfully developed a collagen fibril based on swim bladder collagen-extracted Bester sturgeon fish. They developed hydrogels based on the double network. The double network hydrogels exhibited excellent mechanical properties that had Young’s modulus in the range of 0.26 to 0.93 MPa, the denaturation temperature based on the DSC curve has been increased even up 90 °C, and it also had good biomechanical performance in vivo. Thus, they suggested that this hydrogel can be utilized as artificial cartilage.

Skin defects can be reconstructed via infection, trauma, scarring, genetic defects, and burns, as well as other diseases. To accelerate the wound-healing process, two main approaches can be used: (1) skin regeneration engineering and (2) wound dressing. These approaches, via protecting the wound against infection and bacteria and also accelerating the time of the three stages of the healing process, help the healing of skin defects [130]. For example, Hu et al. [131] tried to improve the wound-healing process via the first approach. They evaluated the wound-healing activity of marine collagen peptides from the skin of Nile tilapia (*Oreochromis niloticus*) via in vitro and in vivo assays. The marine collagen peptides prepared were composed of polypeptides with the contents with molecular weights of less than 5 kDa accounting for 99.14%. In both in vitro and in vivo assays, collagen extracted from Nile tilapia could enhance the wound-healing process. Instead, Zhou et al. [132] prepared electrospun nanofibers as a wound dressing from tilapia collagen. The adhesion, proliferation, and differentiation of human keratinocytes were promoted by electrospun tilapia collagen, and it could facilitate rat skin regeneration.

Collagen derived from marine species could also be used in other tissue engineering areas such as dental, vascular, and corneal. Wang et al. [133] utilized type I collagen as blood and lymphatic vessels that was extracted from fish scales, modified, and crosslinked with methylation and 1,4-butanediol diglycidyl ether, respectively. In vivo studies under growth factor-free conditions showed a favorable interaction between the collagen sample and the surrounding tissue. Liu et al. [134] investigated the in vitro functionality of tilapia fish collagen for periodontal tissue regeneration. The results of cultured human periodontal ligament cells with hydrolyzed fish collagen were investigated by using osteogenic markers ALP, COL I, RUNX2, and OCN at the gene level, and the production of osteogenic-related proteins revealed favorable cell viability and osteogenic differentiation.

Moreover, marine collagen has been used for corneal tissue regeneration by Krishnan et al. [135]. They used collagen from scales of fish (*L. calcarifer*) to fabricate a scaffold; then, they evaluated its physicochemical, mechanical, and cultural characteristics, and compared it with an ideal replacement for the human amniotic membrane. The morphology of a limbal cell on the fish collagen scaffold at hour 48 showed a rapid epithelial migration, although a similar result of cell migration on the human amniotic membrane was observed after 72 h. According to the growth kinetics data, on day 10, the growth area for the fish collagen scaffold was 425 mm^2^, and for the human amniotic membrane, it was 300 mm^2^. The authors suggested that the scaffold may be applied as a candidate for corneal tissue engineering.

Another application of fish collagen is in the drug delivery system. Nowadays, researchers try to deliver drugs to specific body tissues or organs, thereby reducing or eliminating significant challenges such as poor bioavailability, stability, solubility, and absorption [136]. To improve the bioavailability of allopurinol as a drug for treating gout and high levels of uric acid in the human body, Nguyen et al. [137] prepared a pH-sensitive hydrogel based on collagen from fish scales and carrageenan as a drug carrier. This hydrogel could improve the bioactivity and physical properties of the drug in simulated body fluids. The drug release from the carrier was 1.5 to 6.7 times slower than the control sample. In other work, hydrogel microneedles from fish scales collagen crosslinked were prepared by using a modified low-temperature press method, and ferrous gluconate was loaded into them [133]. They swelled to 340% of their initial mass in phosphate buffer solution (PBS) and released 34.5% of the loaded drug during 24 h. Marine collagen could be used as a wound dressing and drug carrier simultaneously. For example, curcumin was loaded into collagen from fish scales and Hyp methylcellulose nanogel for wound-healing applications [138]. The nanogel containing curcumin revealed a prolonged release profile and a higher contraction value of wound. Table 7 summarizes some recent articles about the biomaterials engineering applications of marine collagen.

## 8. Conclusions and Future Perspective

Sustainable biomass and revolutionary biotechnologies not only are already making a significant change in human life but also provide a perspective to the future of studies in some sections of marine and food sciences, chemical engineering, biotechnology, and pharmaceutical sciences. The large variety of applications of collagen and its important roles in the future of tissue engineering make it a key biopolymer for the health and well-being of humans. Given the high demand for collagen, there is an urgent need to find sustainable and cheaper sources of collagen production and also reduce the use of animals. Marine biomass is increasingly becoming a more attractive source for collagen. However, source-dependent variation in the composition, the low melting temperature of marine collagen, and low denaturation temperatures limit its applications. Hence, understanding the physicochemical and biological properties of marine collagen and extraction and purification methods would immensely help address these issues of marine collagen. In this paper, the structure and stability of marine collagen from vertebrate and invertebrate animals were summarized first. Then, different collagen extraction methods, including acid extraction, pepsin-aided AcOH extraction, deep eutectic solvent extraction, supercritical fluid extraction, extrusion, and ultrasound-assisted extraction, as well as the extraction parameters on collagen yield, such as temperature, time, solvent, and solid-to-liquid ratio were reviewed. Among the different extraction methods, deep eutectic solvent extraction is the promising extraction method for future investigation; however, extraction conditions should be optimized to obtain a higher yield of extraction.

Next, some typical marine collagen characteristic methods, mainly FTIR, SDS-PAGE, chromatographic techniques, and DSC, were discussed. Due to the biocompatibility, water-solubility, safety, biodegradability, anti-microbial activity, and functionality, marine collagen is attractive for biomaterials applications, including wound dressing and healing, drug delivery, therapeutics, and tissue engineering and regeneration.

The future study needs to investigate the physicochemical properties of marine collagen and the involvement of factors such as extraction methods on the properties. Researching novel physical, chemical, and enzymatic changes in the structure of marine collagen have to be put forward to make suitable biomaterials from marine collagen. Furthermore, exploring tools for genetic characterization and adopting marine sources of collage will be a new era of making tunable collagen for tissue engineering and other biomedical applications in the near future. 

## Figures and Tables

**Table 1:** A summary of recent collagen isolation and characterization from different fish (vertebrate animals) and invertebrate animals.

Source	Type of Collagen	Source Tissue	Extraction Conditions and Yield (Y)	Remarks	Ref
Small-spotted catshark (*Scyliorhinus canicula*)	Type I	Skin	T ^1^ = 25 °C Time = 34 h AcOH ^2^ = 1 M Y ^3^ = 61.24%	Maximized recovery of collagen in the first stage of extraction (alkaline pretreatment) obtained at 4 °C, 2 h, and 0.1 M NaOH.	[19]
Rabbitfish (*Chimaera monstrosa*), Small-spotted catshark (*Scyliorhinus canicular*), Lantern shark (*Etmopterus* spp.), Catshark (*Galeus* spp.), Cuckoo ray (*Leucoraja naevus*), Common Atlantic grenadier (*Nezumia aequalis*)	Type I	Skin	AcOH = 0.5 M Y = 20%	The collagen type of rabbitfish was different from those of the other studied species. The beta component is very weak, and the alpha 2 components are hardly seen; there are two bands with a molecular weight between alpha 1 and beta dimmer (136 and 161kDa), respectively. The other species has two alpha chains around 100 kDa and a beta component of about 200 kD.	[20]
Atlantic cod (*Gadus morhua*)	Type I	Skin	T = 4 °C Time = 72 h S/L ^4^ = 1/10 AcOH = 0.5 M Y = not evaluated Purity = 90%	Collagen showed a concentration-dependent effect in metabolism and on cell adhesion of lung fibroblast MRC-5 cells.	[39]
Parang-Parang	Type I	Skin	NaOH = 0.1 M AcOH = 0.5 M Time = 12 h Y = 1.915%		[41]
Eel fish	Type I	Skin	AcOH = 0.5 M T = 4 °C Time = 42 h Y = 4.2%	Extracted collagen was used as blue biomaterials for biomedical applications.	[42]
Eel fish (*Evenchelys macrura*)	Type I	Skin	AcOH = 0.5 M Time = 3 days T = 4 °C Y = 4.7%	Pepsin hydrolysis did not affect the secondary structure of collagen.	[43]
Tuna	Type I	Skin and Scales	AcOH = 0.5 M Time = 48 h T = 4 °C Y = 188 g/kg and 177 g/kg	Type I collagen was extracted from fish skin by using bacterial CP ^5^.	[44]
Atlantic cod (*Gadus morhua*)	Type I	Swim bladder	T = 25 °C AcOH = 0.5 M Pepsin ^6^ = 10% Time = 3 days Y = 11.53%	Extracted collagen showed a typical shear thinning behavior, which could be interesting for further processing to develop biomaterials.	[64]
Catla catla and Cirrhinus mrigala	Type I	Skin, scales, and fins	S/L = 1:15 Time (ASC ^7^) = 24 h Time (PSC ^8^) = 48 h T = 4 °C AcOH = 0.5 M Pepsin = 20 U/g Yield = 13, 9.5 and 13; 11.2, 8.3 and 13.1%,	Gly and alanine were the most abundant amino acids, while tryptophan was absent in all used tissues.	[14]
Giant croaker (*Nibea japonica*)	Type I	Skin	S/L = 1:60 Time = 8.5 h T = 4 °C AcOH = 0.5 M Pepsin = 1389 U/g, Y = 84.85%	FTIR ^9^ analysis revealed that PSC maintains its triple-helical structure.	[65]
Sole fish (*Aseraggodes umbratilis*)	Type I	Skin	AcOH = 0.5 M S/L = 1/8.97 (g/mL) Time = 32 h T = 25 °C Y = 19%	Extracted collagen was in the form of fibrils with irregular linkages.	[66]
Atlantic cod (*Gadus morhua*)	Type I	Skin	CO_2_ Pressure = 50 bar T = 37 °C Time = 3 h Y = 13.8%	Type I collagen extracted had a denaturation temperature of 32.3 °C, which can limit its biomaterial applications	[45]
Tilapia (*Oreochromis* sp.)	Type I	Scales	Double distilled H_2_O extraction T = 25–50 °C Time = 1 h S/L = 1/10 Y = 12.3%	Collagen yields from extruded samples were higher than those from non-extruded samples.	[47]
Hoki (*Macruronus novaezelandiae*)	Type II and minor Type IX and Type XI	Nasal cartilage	T = 8 °C AcOH = 0.2 M Pepsin = 0.1% Time = 24 h	A 90 kDa, highly glycosylated collagen, which has not been identified in any other species, was obtained.	[60]
Tilapia (*Oreochromis mossambicus*)	Type I	Bone	Time = 24 h AcOH = 0.5 M Pepsin = 0.1% S/L = 1/20 Y = 3.5% (ASC) Y = 6.0% (PSC)	Extracted collagen (EDTA-treated fishbone) showed a more integrated secondary structure compared to HCl-treated fishbone extraction.	[54]
*Lutjanus* sp.	Type I	Bone	Y = 4.535%	The collagen isolated from *Lutjanus* sp. bone can be used as a natural anticancer agent.	[55]
*Aristichthys nobilis*	Type I	Bone	1% pepsin electric field strength = 20 kV/cm pulse number = 8	The maximum collagen yield of 16.13 mg/mL was obtained.	[58]
Amur sturgeon (*Acipenser schrenckii*)	Type I and Type II with other minor types	Cartilage	Extraction of SSC: 0.45 M NaCl (0.05 M Tris-HCl, pH 7.5), 1:100 (*w*/*v*), 24 h Extraction of ASC: 0.5 M HOAc, 1:100 (*w*/*v*), 24 h Extraction of PSC: 0.1% (*w*/*v*) pepsin in 0.01 M HCl, 1:100 (*w*/*v*), 48 h Y = 27.04% (ASC) Y = 55.92%(PSC) Y = 2.18% (SCC ^10^)	Collagen was observed as a dense sheet-like film linked by random coiled filaments	[48]
Siberian sturgeon (*Acipenser baerii*)	Type I and Type II	Cartilage	NaOH = 0.1 M AcOH = 0.5 M T = 4 °C porcine pepsin = 1% (*w/w*)	The maximum transition temperature (T_max_) of the ASC and PSC was 28.3 and 30.5 °C, respectively.	[61]
Tilapia and Grey mullet	Type I	Scale	AcOH = 0.5 M Time = 3 days Y = 40% (ASC)	Significant inhibitory activity against all the tested bacteria (*Streptococcus mutans*, *Bacillus subtilis*, *Staphylococcus aureus*, and *Escherichia coli*) and wound-closure ability were observed.	[46]
Tilapia (*Oreochromis niloticus*)	Type I	Skin and Scale	AcOH- = 0.5 M Time = 24 h T = 4 °C pH = 7 Y of Scale = 3.2% Y of Skin = 27.2%	The extracted collagen can be a suitable alternative to land-based mammalian collagen.	[67]
Tropical freshwater carp fish (*C. carpio*)	Type I	Scales	AcOH = 0.5 M Time = 24 h Y = 13.6%	The presence of tryptophan, a rare amino acid in collagen, was observed.	[48]
Squid (*Loligo vulgaris*)	Type I and Type V	Mantle	T = 4 °C AcOH = 0.5 M Pepsin = 0.1% Time = 3 days Y = 5.1% (ASC) Y = 24.2% (PSC)	No cytotoxicity was observed by the collagen extracts.	[68]
Marine sponges (*Axinella cannabina* and *Suberites carnosus*)	Type IV	Tissues	Extraction by an alkaline denaturing homogenization buffer (0.1 M Tris-HCl, pH 9.5, 0.01 M EDTA, 8 M urea, 0.1 M 2-mercaptoethanol) Y = 12.6 and 5%	Low amino acid content for the intercellular collagen results in low thermal stability.	[25]
Jellyfish (*Acromitus hardenbergi*)	Type I, II and III	Bell and oral arms	T = 4 °C AcOH = 0.5 M Time = 1 h Y = 37.08% (Bell) Y = 40.20% (Oral arms)	Collagen exhibited better appearance and instrumental color than collagen extracted by conventional methods, and it was found to be non-toxic in vitro and free of heavy metal contamination.	[24]
Surf clam Shell (*Coelomactra antiquatas*	Type I	Body	T = 4 °C Time = 24 h 50 Mm Tris–HCl, pH 7.0 G/HCl ^11^ = 4M Y = 0.59% (GSC) Y = 3.78% (PSC)	The guanidine hydrochloride soluble collagen had a dense sheet-like film linked by random-coiled filaments and PSC had fine globular filaments.	[26]
Antarctic (*Kondakovia longimana*) and Sub-Antarctic squid (*llex argentines*)		Muscles and skin	T = 4 °C Time = 72 h AcOH = 0.5 M Pepsin = 3 mg/g of sample Y = 1.18% and 3.26%	Collagen exhibited an amino acid profile similar to the one of calf collagen, but it exhibited a less preserved structure, with hydrolyzed portions and lower melting temperatures (24–34 °C).	[69]

^1^ Temperature; ^2^ AcOH; ^3^ Yield; ^4^ Solid–liquid ratio; ^5^ Collagenolytic proteases; ^6^ Pepsin-aided extraction; ^7^ Acid-solubilized collagen; ^8^ Pepsin-solubilized collagen; ^9^ Fourier-transform infrared spectroscopy; ^10^ salt-solubilized collagen; ^11^ Guanidine hydrochloride.

**Table 2:** Summary of the experimental conditions employed for fish collagen isolation using AcOH extraction.

Source of Collagen	Extraction Solvent	Extraction Conditions	Yield (Y)	Reference
Swim bladders of yellowfin tuna	0.5 M AcOH	T = 4 °C Time = 48 h S/L = 1/10	Y = 1.07%	[81]
Scales of seabass	0.5 M AcOH	T = 4 °C Time = 48 h S/L = 1/10	Y = 0.38%	[82]
Grass carp skin	0.5 M AcOH	T = 4 °C Time = 72 h S/L = 1/40	Y = 90%	[74]
Skins of catla and rohu fish	0.5 M AcOH	T = 4 °C Time = 72 h S/L = 1/16	Y = 63% (catla) Y = 46% (rohu)	[83]
Scales and skin of tilapia	0.5 M AcOH	T = 4 °C Time = 24 h S/L = 1/10	Y = 27.2% (skin) Y = 3.2% (scales)	[67]
Cod skins	0.5 M AcOH	T = 4 °C Time = 72 h S/L = 1/10	Y = not evaluated	[35]
Sole fish skin	0.5 M AcOH	T = 25 °C Time = 32 h S/L = 1/9	Y = 19%	[79]
Small-spotted catshark skin	0.5 M AcOH	T = 25 °C Time = 34 h	Y = 61.24%	[19]
Catfish (*Ictalurus punctatus*) skin	AcOH, HCl, citric acid, and lactic acid	pH = 1.8, 2.1, 2.4, 2.7 and 3.0 Time = 60 h T = 4 °C	Y = 5% to 42.36%	[78]
Tilapia (*Oreochromis niloticus*) Skin and Scale	0.5 M AcOH	Time = 24 h T = 4 °C pH = 7	Y of scale = 3.2% Y of skin = 27.2%	[84]
Tuna skin, scale, and bone	0.5 M AcOH	Time = 3 days T = 4 °C	Y of skin = 13.5% Y of scale = 0.05% Y of bone = 0.1%	[64]
Sardinella longiceps (*oil Sardine*) Scale	0.5 M AcOH	Time = 4 days T = 4 °C S/L = 1/9	Y = 1.25%	[85]

**Table 3:** Summary of the experimental conditions employed for fish collagen isolation using pepsin aided AcOH extraction.

Source of Collagen	Extraction Solvent	Extraction Conditions	Yield (Y)	Ref
Thornback ray skin	0.2 M AcOH	T = 4 °C Time = 18 h S/L = 1/10 5 g of pepsin/g of skin	Y = 30.16%	[89]
Scales of seabass	0.5 M AcOH	T = 4 °C Time = 48 h S/L = 1/10 20 g of pepsin/g of skin	Y = 1.06%	[82]
Jellyfish	0.6 M AcOH	T = 4 °C Time = 72 h S/L = 1/10 1% pepsin	Y = 0.28%	[90]
Skins of catla and rohu fish	0.5 M AcOH	T = 4 °C Time = 48 h S/L = 1/60	Y = 69% (catla) Y = 65% (rohu)	[83]
Skin of giant croaker	0.5 M AcOH	Pepsin concentration = 800–2400U/g S/L = 1:45–1:65 Time = 6–10 h pH = 1 to 4 T = 4 °C	Y = 84.85%	[87]
By-products of bigeye tuna	0.5 M AcOH	T = 4 °C Time = 48 h S/L = 1/40 0,2 g of pepsin/g of material	Y of bone = 2,6% Y of scale = 4,6% Y of skin = 16,7%	[64]
Cod swim bladders	0.5 M AcOH	T = 25 °C Time = 3 days S/L = 1/10 10% pepsin	Y = 11.53%	[91]
Catfish skin	HCl	pH = 2.4 S/L = 1/5 to 1/20 Pepsin concentration = 0.118 to 23.6 KU/g T = 4 °C	Y = 59.03%	[78]
Nilem fish skin	0.5, 0.7, and 0.9 M AcOH	Pepsin concentration = 0.5, 1, and 1.5% T = 4 °C	Y = 4.25–6.18%	[88]
Silver carp (*Hypophthalmichthys molitrix*) scales	0.5 M AcOH	T = 4 °C S/L = 1/10–1/50 Time = 10–60 h 1–5% Pepsin	Y = the maximum yield 12.06%	[92]
Lophius litulon skin	0.5 M AcOH	T = 4 °C 1–6% Pepsin	Y = not evaluated	[93]
Golden pompano (Trachinotus blochii) Skin and Bone	0.5 M AcOH	T = 4 °C S/L = 1/40 Time = 48 h	Y of skin = 21.81% Y of bone = 1.25%	[94]
Tilapia skin	0.5 M AcOH	Time = 48 h T = 4 °C 0.5% Pepsin	Y = not evaluated	[95]
Sardinella longiceps (*oil Sardine*) Scale	0.5 M AcOH	Time = 4 days T = 4 °C S/L = 1/15 Pepsin = 40 unit/g of residue	Y = 3%	[85]

**Table 4:** Functional groups present in collagen type I.

Amide Structure	Amide Type	Source of the Signal	Wavenumber (cm^−1^)
** 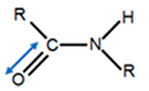 **	I	C=O stretch	1620 < ν < 1800
** 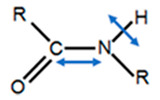 **	II	N–H bend coupled withC–N stretch	1590 < ν < 1650
** 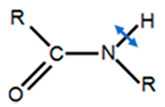 **	III	N–H bend	1200 < ν < 1400
** 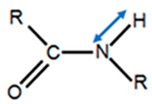 **	A	N–H stretch coupled with hydrogen bond	3300 < ν < 3400

**Table 5:** Expected molar mass ranges for the peptides of collagen type I. The data for this table has been obtained from [35].

Bands of Type I Collagen	Expected Molar Mass (kDa)
α_1_	120–150 kDa
α_2_	120–150 kDa
β_1_	200–250 kDa

**Table 6:** Amino acid distributions of collagen samples obtained from several fish species using different extraction techniques.

Amino Acid	Scales of Seabass (ASC ^1^) [82]	Scales of Seabass (PSC ^2^) [82]	The Skin of Bighead Carp (ASC) [117]	Scales of Bighead Carp (ASC) [117]	The Skin of Nibea Japonica (PSC) [87]
Alanine	133	133	122	118	128
Arginine	52	51	54.7	49.5	51
Asparagine	44	42	48.8	51.9	43
Cysteine	0	0	0.2	0.4	0
Glutamine	71	69	80.3	82.6	73
Glycine	327	337	325	308	348
Histidine	7	7	4.3	4.4	8
Isoleucine	11	9	12.2	12.7	9
Leucine	21	19	23	25.1	25
Lysine	27	26	29.4	26	30
H. Lysine	6	6	2.8	2.4	4.3
Methionine	15	14	16	11.2	10
H. Proline	85	89	66.1	93.6	75
Proline	108	106	115	112	116
Serine	28	33	34.7	31.9	29
Threonine	24	24	0	0	20
Tyrosine	5	3	3.4	3.7	3
Valine	22	20	21.5	22.1	19

^1^ Acid-soluble collagen; ^2^ Pepsin-soluble collagen.

**Table 7:** A summary of fish collagen applications for biomaterials engineering.

Collagen Source	Type of Collagen	Application	Remarks	Ref
Blue shark cartilage	Type II	Bone tissue regeneration	The stiffness increased from 4.71 MPa for collagen scaffold to 8.95 MPa for collagen–hydroxyapatite. The composite sample showed the highest ALP activity	[126]
Fish scale and skin		Bone tissue regeneration	Fish collagen and hydroxyapatite-reinforced poly(lactide-co-glycolide) fibrous membrane had higher stiffness and favorable cytocompatibility with bone mesenchymal stem cells	[127]
Swim bladder	Type I	Bone tissue regeneration	Self-assembled collagen fibrils from the swim bladder improved osteogenic differentiation. The ALP activity increased on day one, while on day five, it decreased	[139]
*Aplysina fulva*		Bone tissue regeneration	Incorporation of collagen from marine sponges (Spongin) into hydroxyapatite samples can be used for bone regeneration application	[140]
Swim bladder	Type I	Cartilage and bone tissue regeneration	Swim bladder collagen-based tough double network hydrogels potential biomaterials as load-bearing implants	[129]
*Lates calcarifer* scale		Bone tissue engineering	A porous scaffold by using fish scale collagen, hydroxyapatite, chitosan, and beta-tricalcium phosphate was prepared	[141]
*Sparus aurata*	Type I	Bone tissue engineering	Preparation of biocomposite scaffold for bone tissue engineering with incorporation bioactive fish scale into chitosan	[142]
Flatfish (*Paralichthys olivaceus*)	Type I	Bone tissue engineering	A polycaprolactone/fish collagen/alginate biocomposite scaffold showed a potential for hard regeneration tissue such as bone	[143]
Silver carp skin (*Hypophthalmichthys molitrix*)		Bone tissue engineering	Histological analysis showed new bone formation after 8 weeks in silver crap skin collagen combined with xenograft	[144]
Shark skin (*Prionace glauca*)		Bone and hard tissue engineering	Collagen from shark skin (*Prionace glauca*) and calcium phosphates from the teeth of two different shark species (*Prionace glauca* and *Isurus oxyrinchus*) were combined and prepared 3D composite scaffold	[145]
Tilapia skin	Type I	Biomedical scaffold for tissue engineering	Fish skin collagen microfiber matrix scaffolds were highly biocompatible and feasible for the development of scaffolds in tissue engineering	[146]
Jellyfish	Type II	Cartilage tissue engineering	Type II collagen from the jellyfish implant leads to the differentiation of mesenchymal stem cells. Therapeutic TGF-β3 as nanoreservoirs that were combined lead to cartilage differentiation	[128]
Antarctic squid Kondakovia longimana skin	Type I	Tissue engineering	Incorporation of extracted collagen from Antarctic squid Kondakovia longimana on poly-ε-caprolactone 3D printed scaffolds for tissue engineering applications	[65]
Jellyfish (Rhopilema esculentum)		Cartilage tissue engineering	Combined jellyfish collagen with alginate for superior chondrogenesis of hMSC	[147]
Nile tilapia (*Oreochromis niloticus*) skin	Type I	Skin regeneration and wound healing	Polypeptides extracted from Nile tilapia skin enhanced wound-healing process through in vitro and in vivo assays	[131]
Tilapia skin	Type I	Wound dressing	The electrospun tilapia collagen nanofibers as a wound dressing could accelerate skin wound healing	[132]
Seawater cultured Tilapia		Skin regeneration	Chitosan hydrogel in combination with marine peptides from tilapia showed antibacterial activity, pro-cell proliferation, and migration, well-burning healing	[148]
Tilapia		Wound dressing	Electrospun fish collagen/bioactive glass nanofibers showed improved skin regeneration with adequate tensile strength and antibacterial activity	[149]
Sponge C. reniformis		Skin regeneration and wound healing	A significant antioxidant activity, no toxicity, and increasing of fibroblast and keratinocytes proliferation have been reported	[150]
Tilapia and grey mullet scale	Type I	Wound healing	All the extracted collagen have inhibitory activity against all of the tested bacteria and also had better closure of the wound	[50]
Nile tilapia skin (*Oreochromis niloticus*)	Type I	Wound dressing	Mechanical strength increased with increasing pepsin soluble collagen in the hydrogel. Hydrogel accelerates the healing of second-degree burn wounds.	[151]
Lophius litulon skin	Type I	Wound healing	In vitro antioxidant study revealed extracted collagen had scavenging ability for 2,2-diphenyl-1-picrylhydrazyl (DPPH), HO·, O2−, and ABTS. The collagen could help ulcer healing due to its compatibility	[93]
Pinctada martensii mantle		Wound healing	The molecular weight of polypeptides extracted from Pinctada martensii was 302.17–2936.43 Da. Small polypeptides molecules promote the proliferation of fibroblasts and keratinocyte	[152]
Jellyfish Rhopilema esculentum	Type I	Wound healing	Protein fragments with molecular weight <25 kDa. Re-epithelialization, tissue regeneration, and increased collagen deposition were improved in histological assessment	[153]
Arothron stellatus fish skin		Wound dressing	Film scaffold based on collagen from Arothron stellatus fish skin and bioactive extract obtained from Coccinia grandis and drug ciprofloxacin Cell adhesion and proliferation of the film sample was higher than the control sample	[154]
Tilapia		Wound healing	Tilapia collagen was mixed with TY001 as a promotive healing process. Increase of insulin growth factor-1, basic fibroblast growth factor, platelet-derived growth factor, transforming growth facts β 1, vascular endothelial growth factor, and epidermal growth factor	[155]
Snakehead scales	Type I	Vascular tissue engineering	Good infiltration of cells, blood vessels, and lymphatic vessels were showed by collagen extracted from fish scales	[133]
Tilapia scale	Type I	Oral mucosa tissue	Histologic evaluation illustrated that all scaffolds based on the microstructured fish collagen have the potential for use in oral mucosa tissue	[156]
Tilapia		periodontal tissue regeneration	The results of osteogenic markers, including ALP, COL I, RUNX2, and OCN showed cell viability and osteogenic differentiation	[134]
Sish scale (*L. calcarifer*)		Corneal Tissue Engineering	At day 15, 90 to 100% confluent growth showed similar morphological features of limbal epithelium	[135]
Carp fish scales	Type I	Drug delivery	The stability of the drug was increased, and also the release was slower than the control sample	[137]
Fish scales		Drug delivery	The microneedles hydrogels released 34.5% of drug-loaded during 24 h	[133]
Fish scales		Drug delivery and wound dressing	Curcumin was loaded into nanogel-based fish scale collagen for delivery of the drug to the wound.	[138]
Fish scales		Drug delivery and wound dressing	Fish scales collagen film was used to release aspirin. The concentration of aspirin after 48 h from microneedles hydrogel was 0.74 mg/mL	[157]

## Abbreviations

AcOH	Acetic acid
ALP	Alkaline phosphatase activity
ASC	Acid-soluble collagen
BSE	Bovine spongiform encephalopathy
CC	Choline chloride
CP	Bacterial collagenolytic proteases
DES	Deep eutectic solvent
DSC	Differential scanning calorimetry
ECM	Extracellular matrix
EHE	Extrusion-hydro-extraction
EDTA	Ethylenediaminetetraacetic acid
FTIR	Fourier transform infrared spectroscopy
Gly	Glycine
HBA	Hydrogen bond acceptor
HBD	Hydrogen bond donor
HCl	Hydrochloric acid
Hyp	4-Hydroxyproline:
NaOH	Sodium hydroxide
PBS	Phosphate buffer solution
PEF	High-intensity pulsed electric fields
PSC	Pepsin-soluble collagen
SBE	Semi-bionic extraction
SDS-PAGE	Sodium dodecyl sulfate sulfate-polyacrylamide gel electrophoresis
SFE	Supercritical fluid extraction
OA	Oxalic acid
